# Epidemiological Investigation of Bovine Ephemeral Fever Outbreaks in Israel

**DOI:** 10.4061/2010/290541

**Published:** 2010-08-15

**Authors:** Israel Yeruham, Michael Van Ham, Yehuda Stram, Orly Friedgut, Hagai Yadin, Kosta Y. Mumcuoglu, Yehuda Braverman

**Affiliations:** ^1^Koret School of Veterinary Medicine, The Hebrew University of Jerusalem, Rehovot, Israel; ^2^The Veterinary Services, Bet Dagan, Israel; ^3^The Kimron Veterinary Institute, Bet Dagan, Israel; ^4^Department of Microbiology and Molecular Genetics, The Kuvin Center for the Study of Infectious and Tropical Diseases, The Institute for Medical Research Israel-Canada, The Hebrew University - Hadassah Medical School, P.O. Box 12272, Jerusalem 91120, Israel

## Abstract

Outbreaks of bovine ephemeral fever (BEF) occurred in Israel in 1990, 1999, and 2004. The main patterns of BEF spread were similar in the 1990 and in 1999 epidemics, and the BEF virus was probably carried in vectors transported by air streams across the Rift Valley and the Red Sea. In the 2004 outbreak, the primary focus of the disease was the southern Mediterranean coastal plain and the disease agent was apparently brought by infected mosquitoes carried from their breeding site in the Nile Delta by the south-western winds. The disease broke out under optimal ecological conditions, among a vulnerable cattle population and spread rapidly; it showed essentially a spring-summer herd incidence and terminated soon after the night average ambient temperature fell below 16°C in late autumn. The herd incidence of the disease reached 78.4%, 97.7%, and 100% in 1990, 1999, and 2004, respectively. The highest herd incidence, morbidity, and case fatality rates were noted in dairy cattle herds in the Jordan Valley, with morbidity of 20%, 38.6%, and 22.2%, and case fatality rate among affected animals of 2%, 8.6%, and 5.4% in 1990, 1999, and 2004, respectively. The average sero-positivity to BEF in 1999 was 39.5%, which matched the morbidity rate. Comparison among the various age groups showed that the lowest morbidity rates were observed in the youngest age group, that is, heifers up to 1 year, with 3.2%, 3.6%, and 4.2% in 1990, 1999, and 2004, respectively. In heifers from 1 year to calving, the morbidity rates were 13.8%, 14.9%, and 28%, respectively, in first calvers 30.8%, 31.6%, and 28.3%, respectively, and in cows 34.3%, 35.7%, and 27.2%, respectively. All affected cattle were over the age of 3 months. It is hypothesized that mosquitoes and not *Culicoides* spp. are the vectors of the BEF virus in Israel.

## 1. Introduction

Bovine ephemeral fever (BEF), caused by the bovine ephemeral fever virus (family Rhabdoviridae, genus *Ephemerovirus)* is a noncontagious inflammatory disease, of short duration, that affects cattle [1]. The BEF virus life-cycle is maintained through a vector-host system [2]. BEF is not transmitted by close contact, bodily secretions, or aerosol droplets, and carriers are not known to occur [3, 4]. The virus agent has been isolated from various species of midges and mosquitoes, which are probably the main vectors [5, 6]. BEF is spread by movement of the host or by vectors [2, 4], but long-distance carriage of infected insects by the wind has most likely been responsible for the spread of BEF in various countries [4].

According to Sellers [7], Israel and the rest of the Mediterranean area are in zone “C”, in which pathogens are introduced by infected vectors carried on warm winds. Outbreaks of BEF occur when vector—probably mosquito—populations increase, resulting in high rates of virus transmission to susceptible cattle.

There is some evidence to suggest that BEF is immune mediated in nature [8] and that the clinical characteristics of the disease are the expression of mediators of inflammation common to a number of acute febrile diseases with a secondary hypocalcaemia [3, 4]. Clinical signs and pathological changes are reflections of the effect of the growth of the virus and the host's response [8].

BEF was first described in 1924 in Egypt (Rabagliati 1924, cited by Sen [9]) and in the Jordan Valley in Palestine in 1931 [10]. Since then the disease has occurred at irregular, long intervals, and the last outbreaks, previous to those discussed in the present paper, occurred in 1951. The disease has also been reported in Jordan, Syria, Iraq, Iran, and Saudi Arabia [8, 11].

The present paper presents an analysis of the data on BEF outbreaks in Israel available from the 1990, 1999, and 2004 epizootics. The 2008 epizootic of BEF in Israel was also noted.

## 2. Materials and Methods

### 2.1. Geography and Climate

Apart from the herds located in the Jordan Valley, which is part of the Irano-Turanian (northern part), Saharo-Arabian and Ethiopian (southern parts) zoogeographical zones, all other herds were located in the Mediterranean zoogeographical zone [12]. The appropriate weather charts were obtained from the Israel Meteorological Service at Bet-Dagan and from the Climatic Atlas of Israel for Physical and Environmental Planning and Design [13], and the publications of Jaffe [14] and Goldreich [15]. 

The Jordan Valley forms a part of the lee-side dry lands to the east of the hill region. Those regions are semi-arid with mean annual precipitation of 270 mm, while in the Mediterranean zoogeographic zone the climatic conditions with a mean annual precipitation of 550 mm are more humid.

### 2.2. The Herds

The 192 cattle herds included in this survey comprised 97,564 animals. They were located in three regions where the BEF epidemic occurred, that is, the Jordan Valley (33°05'N, 35° 07'E to 31° 45'N, 35° 27'E), the inner valleys (32° 42'N, 35° 33'E to 32° 34'N 35° 11'E), and the Mediterranean coastal plain (33° 05'N, 35° 7'E to 32° 13'N, 34° 56'E).

The cattle in the dairy herds (Holstein-Israeli) were kept under a zero-grazing management system, and gave an average milk yield of 11,000 to 12,000 1/yr. Accurate epidemiological and clinical data relating to the incidence of morbidity and case fatality rate were recorded by computerized dairy management systems. Morbidity and case fatality rate are within herd values, whereas herd incidence refers to the proportion of herds that were affected. Only cattle that exhibited clinical signs consistent with BEF infection were included in the morbidity data, and those that succumbed to the infection were included in the case fatality rate analyses. The cause of death was confirmed by pathological examination of randomly selected carcasses.

### 2.3. Serology

A serological survey was conducted once in the 1999 and a second time in the 2000 outbreak. In the 1999 outbreak, 25 naturally infected dairy herds out of the 48 in the main survey served as an indicator of the prevalence of BEF virus activity. Forty blood samples were collected during February-March 2000 from randomly selected cattle in each herd, in various age groups: nine samples from heifers up to 1 year of age, eight samples from heifers up to calving, six samples from first-calvers, and 17 samples from mature cows. 

BEF infection elicits neutralizing antibodies in sera of infected cattle. The antibody titers were assessed by a serum neutralization test (SN) in a microassay system of Vero cells cultured against 100 TCID50 of the BEF virus strain BB7721 passage 12 on BHK21. All sera were titrated in duplicates in a 2-fold dilution series, with an initial dilution of 1/4. The plates were read for CPE under the microscope after 96 h or stained with 0.2% Amidoblack in methanol. The SN titer was expressed as the negative log of the highest serum dilution. Serology provided a presumptive confirmation of infection in the first outbreak and in the younger animals in subsequent outbreaks; however, it is possible that the older animals in later outbreaks may have been previously infected.

## 3. Results

Three major outbreaks have been observed: in 1990, 1999, and in 2004. The outbreaks of 1990 and 1999 were each followed by another episode in the following year [16]. The major epizootics of BEF in 1990, 1999, and 2004 commenced in September, May, and June, respectively, and terminated in November (1990, 2004) and December (1999). The clinical signs were consistent with BEF virus infection. In the epidemics of 1990 and 1999, the disease started in the north of the Jordan valley spread northwards along the Jordan Valley and southwards to the Dead Sea area; while later it advanced westwards to the inner valleys. In the 1999 epidemic, the disease continued to spread even further westwards and southwards along the Mediterranean coastal plain.

In 2004, the disease spread from the primary focus in the southern part of the Mediterranean coastal plain, northwards along the Mediterranean shore, as well as eastwards and southwards. Only cattle over the age of 3 months were clinically affected. The herd incidence average rate of the disease in the affected areas reached 78.4% (73 herds), 97.7% (170 herds), and 100% (192 herds) in 1990, 1999, and 2004, respectively.

The highest morbidity and case fatality rates were noted among dairy cattle herds located in the Jordan Valley: morbidity and case fatality rates among the affected animals reached 20% and 2%, respectively, in 1990, 38.6% and 8.6%, respectively, in 1999, and 22.25 and 5.45, respectively, in 2004. The rate of sero-positive cattle was 39.5% in herds examined during the 1999 outbreak, and in the Jordan Valley was almost identical to the morbidity rate (38.6%). The morbidity period in this region in 1990 was relatively short (September to November) compared with that of the epidemic in 1999 (May to December), while in 2004, the morbidity period (September to November) was similar to that of 1990. The disease terminated soon after the night average ambient temperature decreased below 16°C [17].

In the 1990 outbreak, the morbidity rate ranged from an average of 20% in the Jordan Valley to 8.3% in the inner valleys, and a similar pattern was also observed in the 1999 outbreak, when the average morbidity rate decreased considerably, from 38.7% in the Jordan Valley to 5.4% on the Mediterranean coastal plain, whereas in the 2004 outbreak the average morbidity rate increased from 14.9% in the primary focus in the southern coastal plain to 22.2% in the Jordan Valley. The case fatality rate in 1999 increased from 8.6% in the Jordan Valley to 28% in the Mediterranean coastal plain, and in 2004 it increased from 3.5% in the southern coastal plain to 5.4% in the Jordan Valley.

The morbidity rates in heifers up to 1 year old reached 3.2%, 3.6%, and 4.2% in 1990, 1999, and 2004, respectively. In first calvers, the morbidity rates were similar in all three outbreaks, and reached 30.8%, 31.6%, and 28.3%, respectively. In cows the morbidity rates in 1990 and 1999 were similar, at 34.3 and 35.7%, respectively, while in 2004 decreased to 27.2%.

The case fatality rate patterns in 1990 and 2004 were similar; the lowest, in young heifers (up to 1 year old) were 1.6% to 1.9%, respectively, and the highest, in cows, were 8.5% in 1990 and 6.3% in 2004. The case fatality rates in all age groups were higher in the 1999 outbreak, ranging from 8.8% in heifers aged from 1 year to calving up to 11.5% in cows.

## 4. Discussion

The outbreak of 1999 in the Jordan Valley was more severe (with morbidity and case fatality rates of 38.6% and 8.6%, resp.) than that in 1990 (with morbidity and case fatality rates of 20% and 2%, resp.) and 2004 (with morbidity and case fatality rates of 22.2% and 5.4%, respectively).

The morbidity and case fatality rates in the 1999 outbreak were higher than have been reported elsewhere, for example, Saudi Arabia and Australia [4, 11, 18]. The morbidity rates in the 1990 and 1999 outbreaks diminished with increasing distance from the primary focus of BEF in the Jordan Valley [19], a pattern that is similar to the situation reported in Australia [20] and China [21], with a grading from the tropical zone in the Jordan Valley to the Mediterranean zone in the coastal plain.

An opposite pattern occurred in the 2004 outbreak—the morbidity rate was lower in the primary focus of the disease in the southern part of the Mediterranean coastal plain (14.9%) and higher in the Jordan Valley (22.2%). It seems that, in addition to a susceptible cattle population and a low level of herd immunity to the BEF virus, other factors in the Jordan Valley provided optimum conditions for the propagation and dispersal of the disease.

The higher morbidity and case fatality rates in the 1999 outbreak indicate that the cattle population was susceptible to BEF virus, and probably that the virus strain involved in the 1999 epidemic was highly virulent. Interestingly, all the cattle affected were over the age of 3 months. This may indicate that calves younger than 3 months possess natural resistance to the BEF virus [16, 19].

The morbidity rate of adult cows in the 2004 outbreak was lower (27.2%) than those in the previous outbreaks in 1990 (34.3%) and in 1999 (35.7%). It seems that the mature cattle population possessed some degree of naturally immunity and resistance to BEF. The hypothesis of St George [4] that one infection usually confers lifelong immunity seems to be valid also in Israel. The morbidity rate in the Jordan Valley in 1999 (38.6%) matched the serologically determined herd infection rate of 39.5%.

Climatic and environmental factors govern the survival and distribution of insect vectors and of the pathogens they may transmit [22]. The role of mosquitoes in the perpetuation and dissemination of the BEF virus has been described [4, 23]. Mosquitoes such as* Culex pipiens* and *Ochlerotatus caspius* and several species of *Culicoides *midges, including *Culicoides imicola, Culicodes kingi, Culicoides oxystoma* (the last two species belongs to the *schultzei* group), and *Culicoides punctatus,* which are known vectors of arboviruses that infect animals, have been identified in areas in Israel where BEF has occurred [24].

The fact that the outbreak of 1999 started early in the season, that is, in April, is not typical of diseases vectored by *Culicoides*; these typically start in July and are associated with the air current of the Persian trough [25]. Most probably it was the mosquito population which reached the critical density and facilitated the early transmission in the hot Jordan Valley than in the cooler coastal plain and, therefore, BEF outbreaks started in the spring in the hotter regions [24]. 

These arguments support the hypothesis that BEF virus was probably transmitted by mosquitoes and not by *Culicoides* spp. It seems that the eastern basin of the Mediterranean is a fringe region of the geographical distribution of BEF, into which the disease makes irregular but often spectacular spring and summer incursions [25, 26]. 

The spread of the disease apparently followed the prevailing winds in these regions during the BEF morbidity period [17]. Retrospective analysis of the spreading patterns of the epidemics of 1990 and 1999 found similarities which could indicate that vectors infected with BEF virus could have been brought into the Jordan Valley on seasonal winds blowing over relatively long distances from eastern or southern regions where the disease is endemic [11, 27, 28]. The Red Sea trough is probably responsible for the carriage of infected vectors in the spring and late autumn [29].

During the summer months of 1990, BEF occurred in several regions of Saudi Arabia, where the disease began in May [30], and this was probably the source of the BEF virus in our region. The 1999 epidemic in Israel also started on May; however, no information on BEF morbidity in neighboring countries has been reported. Between 1991 and 1999 and from 2001 to 2004, Israel remained free from BEF infection.

The primary source of BEF infection in the 2004 outbreak in Israel was the southern coastal plain, which is located 180–200 km from the Nile Delta. The Nile Delta offers favorable conditions for breeding large population of biting flies, mainly mosquitoes, and it is known to be a permanent focus of BEF infection. Such a distance could easily be crossed by infected mosquitoes carried with the wind [28]. The carriage of flies over long distance by air currents has also been reported by Farag et al. [30], Braverman and Chechik [29], Polydorou [31], Ward [32], and A1ba et al. [33]. It was reported that *Anopheles pharoensis*, a known malaria vector, was blown 180 km from the Nile delta in Egypt [34] to Tantura (=Dor) at the northern coastal plain of Israel [35]. 

Analysis of the meteorological conditions that prevailed on the days before the onset of the BEF outbreak (wind speed and direction, mild temperature and high humidity) and consideration of the incubation period and the duration of the viremia in cattle, as well as the eclipse phases of the BEF virus in the vectors, suggest that airborne transport of mosquitoes was the most likely way by which the BEF virus was introduced to the southern Mediterranean coastal plain, where the 2004 BEF epidemic started.

The rapid spread of the BEF epidemics from the primary foci was most probably supported by the dense populations of susceptible cattle, breeding places of vectors, and climatic and ecological conditions suitable for the propagation and dispersal of massive numbers of vectors. Probably the cattle which circulated the virus in their peripheral blood infected a large number of vectors and so contributed considerably to the fast spread of the disease [36]. 

There is no evidence that BEF could be transmitted from cow to cow by direct contact [37]. Mechanical transmission via insect vectors seems improbable, and the virus does not persist much beyond the 4th day after subsidence of the fever [3]. Thus, the BEF virus is unlikely to be maintained by subclinical infections in cattle during interepizootic periods, as was postulated by St George et al. [38]. No evidence of carrier animals has been found experimentally or suspected from epidemic outbreaks [37].

The major factor contributing to the abatement of the Israeli epidemics was the onset of cold weather in December, when the night average ambient temperature decreases below 16°C, a level which suppresses vector activity and is not conducive to virus transmission [39]. It was hypothesized that the BEF virus transmitted transovarially by the mosquito vector [4, 40]. Apparently the insect vectors play a crucial role both in the transmission of the BEF virus and also as a reservoir during the winter months and during interepizootic periods. This mode of transmission maintains the biological cycle of the BEF virus, so that the continuous presence of adult vectors seems to be essential for virus persistence [5].

In spite of the above-mentioned favorable conditions, BEF epidemics in this region appear to have an unpredictable pattern. The occurrence of epidemics separated by long intervals most probably resulted in a cattle population that is highly susceptible to BEF. The outbreaks occurred between April and December, when the climate in this particular region is most favorable to the existence of a critical mass of vectors. That is in accordance with the observations of Davies et al. [41] in Kenya, where there was an association between epidemics of BEF and prolonged rainfalls that resulted in a large population of mosquitoes.

The occurrence of the BEF epidemics at short intervals in the last decade was probably caused by for example, the creation of new irrigation systems and dams, which provided more breeding sites to the mosquito vectors. Due to higher standard of living, more cattle are raised and imported to Israel, and accordingly more reservoir hosts are available for the virus. Other potential factors that could influence the occurrence of BEF outbreaks include viral incursions into surrounding countries and the introduction of new viral strains.

Since the completion of this study in 2004, a new outbreak of BEF occurred in September–November 2008, where cattle herds in 90 localities were affected. It started in the western Galilee, moved to the lake of Galilee and Golan Heights, and later spread further to south and north of the Jordan valley as well as westwards to Afula and Hadera [42]. 

In the four epidemics, which were observed in Israel, once the disease has been confirmed, the movement of cattle from infected regions was banned. Treatment with nonsteroidal anti-inflammatory and supportive medication was given. Spraying or pour-on treatment of the animals with repellents was also recommended.

## References

[1] Uren MF, St. George TD, Murphy GM (1992). Studies on the pathogenesis of bovine ephemeral fever in experimental cattle III. Virological and biochemical data. *Veterinary Microbiology*.

[2] Murray MD (1997). Possible vectors of bovine ephemeral fever in the 1967/68 epizootic in northern Victoria. *Australian Veterinary Journal*.

[3] Nandi S, Negi BS (1999). Bovine ephemeral fever: a review. *Comparative Immunology, Microbiology and Infectious Diseases*.

[4] St George TD (1988). Bovine ephemeral fever: a review. *Tropical Animal Health and Production*.

[5] Mellor PS (1996). Culicoides: vectors, climate change and disease risk. *Veterinary Bulletin*.

[6] Venter GJ, Hamblin C, Paweska JT (2003). Determination of the oral susceptibility of South African livestock-associated biting midges, *Culicoides* species, to bovine ephemeral fever virus. *Medical and Veterinary Entomology*.

[7] Sellers RF (1980). Weather, host and vector—their interplay in the spread of insect-borne animal virus diseases. *Journal of Hygiene*.

[8] Burgess GW (1971). Bovine ephemeral fever: a review. *Veterinary Bulletin*.

[9] Sen SK (1931). Three-day sickness of cattle. *Indian Journal of Veterinary Science*.

[10] Rosen SG (1931). Ephemeral fever of cattle in Palestine. *Veterinary Journal*.

[11] Abu Elzein EME, Gameel AA, Al Afaleq AI, Al Gundi O, Bukhari A (1997). Bovine ephemeral fever in Saudi Arabia. *Veterinary Record*.

[12] Theodor O, Costa M (1967). *A Survey of the Parasites of Wild Mammals and Birds in Israel. Part I. Ectoparasites*.

[13] Bitan A, Rubin S (1994). *Climatic Atlas of Israel for Physical and Environmental Planning and Design*.

[14] Jaffe S, Yom-Tov Y, Tchernov E (1988). Climate of Israel. *The Zoogeography of Israel*.

[15] Gold Y (1998). *The Climate of Israel, Observations, Research and Application*.

[16] Yeruham I, Gur Y, Braverman Y (2007). Retrospective epidemiological investigation of an outbreak of bovine ephemeral fever in 1991 affecting dairy cattle herds on the Mediterranean coastal plain. *Veterinary Journal*.

[17] Gat Z, Karni O (1995). *Climate and Agrometeorology of the Jordan Valley, Adjacent Samaria Slopes and Dead Sea Regions as a Basis for Agricultural Planning and Operation*.

[18] Newton LG, Wheatley CH (1970). The occurrence and spread of ephemeral fever of cattle in Queensland. *Australian Veterinary Journal*.

[19] Yeruham I, Braverman Y, Yadin H (2002). Epidemiological investigations of outbreaks of bovine ephemeral fever in Israel. *Veterinary Record*.

[20] Uren MF, St George TD, Kirkland PD, Stranger RS, Murray MD (1987). Epidemiology of bovine ephemeral fever in Australia 1981–1985. *Australian Journal of Biological Sciences*.

[21] Wenbin B, Chunling J, Davis SS (1991). Preliminary observations on the epidemiology of bovine ephemeral fever in China. *Tropical Animal Health and Production*.

[22] Thomson MC, Connor SJ (2000). Environmental information systems for the control of arthropod vectors of disease. *Medical and Veterinary Entomology*.

[23] Kaneko N, Inaba Y, Akashi H, Miura Y, Shorthose J, Kurashige K (1986). Isolation of a new bovine ephemeral fever group virus. *Australian Veterinary Journal*.

[24] Braverman Y The vectors of bovine ephemeral fever, Akabane and bluetongue viruses in Israel.

[25] http://www.ivis.org/advances/disease-factsheets/bovine_ephemeral_fever.pdf?LA=1.

[26] http://www.promedmail.org/.

[27] Abu Elzein EME, Gameel AA, Al-Afaleq AI (1999). Observations on the recent epizootic of bovine ephemeral fever in Saudi Arabia. *Revue Scientifique et Technique*.

[28] Al-Busaidy SM, Mellor PS (1991). Isolation and identification of arboviruses from the Sultanate of Oman. *Epidemiology and Infection*.

[29] Braverman Y, Chechik F (1996). Air streams and the introduction of animal diseases borne on *Culicoides* (Diptera, Ceratopogonidae) into Israel. *Revue Scientifique et Technique*.

[30] Farag MA, Al-Sukayran A, Mazloum KS, Al-Bukomy AM (1998). Epizootics of bovine ephemeral fever on dairy farms in Saudi Arabia. *Revue Scientifique et Technique*.

[31] Polydorou K (1980). The epizootiology, diagnosis and control of Bluetongue in Cyprus. *Bulletin de l’Office International des Epizooties*.

[32] Ward MP (2000). Forecasting blowfly strike in Queensland sheep flocks. *Veterinary Parasitology*.

[33] Alba A, Casal J, Domingo M (2004). Possible introduction of bluetongue into the Balearic Islands, Spain, in 2000, via air streams. *Veterinary Record*.

[34] Garrett-Jones C (1962). The possibility of active long distance migrations by *Anopheles pharoensis* Theobald. *Bulletin of the World Health Organization*.

[35] Saliternik Z (1960). Malaria and *Anopheles* mosquitoes in the Mediterranean coastal plain in 1959. *Briut Hazibur*.

[36] Birkett MA, Agelopoulos N, Jensen K-MV (2004). The role of volatile semiochemicals in mediating host location and selection by nuisance and disease-transmitting cattle flies. *Medical and Veterinary Entomology*.

[37] Mackerras IM, Mackerras MJ, Burnet FM (1940). *Experimental Studies of Ephemeral Fever in Australian Cattle*.

[38] St. George TD, Standfast HA, Christie DG, Knott SG, Morgan IR (1977). The epizootiology of bovine ephemeral fever in Australia and Papua-New Guinea. *Australian Veterinary Journal*.

[39] Braverman Y, Rechtman S, Frish A, Braverman R (2003). Dynamics of biting activity of *C. imicola* kieffer during the year. *Israel Journal of Veterinary Medicine*.

[40] Kirkland PD Bovine ephemeral fever in the Hunter Valley of New South Wales 1927–1981.

[41] Davies FG, Ochieng P, Walker AR (1990). The occurrence of ephemeral fever in Kenya, 1968–1988. *Veterinary Microbiology*.

[42] Belaish M (2008). *2008th Annual Report*.

